# Retrospective Evaluation of Maxillary Sinus Volume Changes Following Bone-Anchored Midface Distraction Osteogenesis in Cleft Patients: A Case–Series Study

**DOI:** 10.3390/jcm14207422

**Published:** 2025-10-21

**Authors:** Aleksandra Kołodziejska, Patryk Kołodziejski, Maria Gutowska, Martyna Dowgierd, Agnieszka Predko-Engel, Monika Jurczuk, Krzysztof Dowgierd

**Affiliations:** 1 Division of Orthodontics, Medical University of Gdańsk, Al. Zwycięstwa 42c, 80-210 Gdańsk, Poland; aleksandra.chorzewska@gumed.edu.pl; 2 Orthodontic Department, Regional Specialist Children’s Hospital, Ul. Żołnierska 18a, 10-561 Olsztyn, Poland; 3 Department of Maxillofacial and Reconstructive Surgery for Children and Adolescents Olsztyn, Regional Specialist Children’s Hospital, Ul. Żołnierska 18a, 10-561 Olsztyn, Polandkrzysztofdowgierd@gmail.com (K.D.); 4 Private Practice Stomatologia Predko-Engel, Ul. Jerzego Waszyngtona 32/lok. 19, 15-304 Białystok, Poland; 5 Faculty of Medicine, Clinic of Head and Neck Surgery for Children and Adolescents, Department of Clinical Pediatrics, University of Warmia and Mazury in Olsztyn, Ul. Żołnierska 18a, 10-561 Olsztyn, Poland

**Keywords:** maxillary sinus, osteogenesis, cleft lip, cleft palate

## Abstract

**Background/Objectives**: This study retrospectively evaluated maxillary sinus volume changes and linear changes in the craniofacial region after Le Fort I distraction osteogenesis using a rigid external distraction system. **Methods**: Ten patients who underwent LeFort 1 distraction osteogenesis between 2012 and 2025 were included in the study. Computed tomography scans and lateral cephalograms were obtained before and 12.3 ± 6.98 months after the surgery. The associated volumes of the maxillary left and right sinuses were subsequently measured using the semiautomatic segmentation method in the ITK-SNAP software. Linear measurements of the sinuses and cephalometric analysis were performed before and after the distraction. **Results**: The Wits appraisal (distance between perpendicular lines drawn from point A (on the maxilla) and point B (on the mandible) to the occlusal plane) presented an increase of 9.33 mm ± 7.93 mm, corresponding to an increase in the ANB angle by 9.88° ± 5.35°. There were statistically significant increases in the total sinus volume, by 3965 mm^3^ ± 5456 mm^3^ (n = 10, *p* = 0.047), and in the single maxillary sinus volume, by 1983 ± 2889 mm^3^ (n = 20, *p* = 0.003). A significant increase in height was also observed, with a mean value of 4.46 ± 2.94 mm (n = 20). **Conclusions**: Extraoral bone-anchored midface distraction osteogenesis led to increases in single sinus volume, total sinus volume and sinus height in the cleft cohort, resulting in improved maxillary retrusion and profile. However, the study group was small and non-uniform with different follow-up periods, indicating a need for further studies with larger, more homogenous cohorts.

## 1. Introduction

The maxillary sinus is a pneumatised region in the maxillary bone. Its development begins in the embryonic period—around the 10th week—from the mucosa of the primitive ethmoidal infundibulum [1], with the most significant growth observed up to the 3rd year of life. After this period, it slows down and accelerates again at around 7–12 years of age. Pneumatisation is usually complete at around 15 years of age, when all the teeth have fully erupted [2]. The paranasal sinuses are assumed to reduce the weight of the skull, strengthen vocal resonance, humidify the inhaled air and contribute to maxillary growth [3]. Significant research has been undertaken to assess whether the presence of a cleft lip/palate influences the growth and development of the maxillary sinus.

Cleft lip and palate is one of the most common craniofacial deformities in the human population [4], which occurs as a result of an interruption during the connection of the primary palatal plates in the embryonic period. According to the European Surveillance of Congenital Anomalies (EUROCAT) data, the incidence of cleft palate together with cleft lip is 5.95–8.91 per 10,000 live births [5].

The introduction of computed tomography (CT) has enabled researchers to accurately measure the maxillary sinus, proving itself to be a precise and reproducible tool in this field [6]. Since then, new technologies have been invented to assess the volume of three-dimensional (3D) images. The 3D segmentation method has been implemented in various software, such as Dolphin, Mimics, ITK-SNAP and others, and can be utilised for assessment of the maxillary sinus volume in cleft lip/palate patients [7]. Three-dimensional segmentation tools have been shown to enable accurate and reliable assessments of the volume of the maxillary sinuses, as well as other regions [8,9,10]. Some studies have indicated that the maxillary sinus volume is diminished in cleft patients [11], while others did not observe such a correlation [12].

Distraction osteogenesis is a surgical method for the treatment of skeletal discrepancies, involving gradual bone lengthening through controlled mechanical traction. It was first introduced in orthopaedic surgery by Codivilla [13] and Ilizarov [14], who laid the foundation for long bone distraction. Craniofacial distraction osteogenesis was first introduced by McCarthy in 1992 [15], and the first reported human midface distractions were performed by Habal et al. [16] and Cohen et al. [17]. In 1994, distraction of the maxillary bone in cleft patients was performed by Molina with the use of reverse headgear. In 1997, Cohen utilised fixed appliances for maxillary distraction in cleft patients [18].

Since then, midfacial distraction osteogenesis has become an established method of stable maxillary advancement in syndromic and non-syndromic patients with severe midface hypoplasia [19,20]. Compared to classic orthognathic procedures, midface distraction facilitates greater advancement and has a lower incidence of relapse. It has been proven that maxillary distraction is safer than classic orthognathic surgery in terms of speech complications, especially for cleft patients [21,22].

While growing interest in the changes in maxillary sinus volume following orthognathic surgeries can be observed in the literature [23,24], there is a paucity of data regarding sinus volume changes following midface distraction, especially in the cleft lip/palate group.

The aim of this study was to evaluate maxillary sinus volume changes and linear changes in the craniofacial region after Le Fort I distraction osteogenesis using a rigid external distraction (RED) system. The null hypothesis was that Le Fort I maxillary distraction osteogenesis does not influence the maxillary sinus volume in the study group. The study was conducted following the Strengthening The Reporting of Observational Studies in Epidemiology (STROBE) guidelines, and was approved by the institutional review board—namely, the Ethics Committee of the Medical University of Gdansk, Poland (Reference no. KB/162/2025, 4 April 2025)—in accordance with the Declaration of Helsinki. Informed consent was obtained from all subjects involved in the study.

This study aims to contribute to a better understanding of sinus changes following midfacial distraction procedures, as well as revealing the effects of the operation on sinus inflammatory disease.

## 2. Materials and Methods

Data were collected from 7 April to 21 April 2025, following the institutional review board’s decision. The hospital’s database was searched from 1 January 2012 to 1 January 2025. The inclusion criteria were as follows: patients aged 0–25 with unilateral or bilateral cleft lip and palate who were treated via maxillary Le Fort I distraction osteogenesis due to maxillary hypoplasia, presented class III malocclusion, and had radiological assessment (CT scans and lateral cephalograms) performed before and after the surgical procedure. The exclusion criteria were as follows: patients with isolated cleft lip or isolated cleft palate, syndromic cleft lip and palate, or other craniofacial deformities; patients treated with intraoral distraction devices; patients with image artefacts; and those with incomplete data records.

The following data were collected: patients’ demographic and clinical characteristics (including age, sex, and cleft type); details of the distraction procedure (osteotomy type, distractor activation period, and extent of distraction); and cephalometric, linear, and volumetric parameters, analysed before and after distraction.

The CT data were stored in Digital Imaging and Communications in Medicine (DICOM) files. Pre- and post-distraction Segner and Hasund cephalometric analyses were performed using the DDP-Ortho software version 3.6.0 (Częstochowa, Poland). All measurements were taken twice, within a two-week interval, by the same researcher with over five years of experience.

Linear measurements were performed on all sinus CT scans before and after the end of the distraction. The measurements were carried out according to the method of Przystańska et al., considering the maximum diameter in three planes [25]. The height of the maxillary sinus (maximal craniocaudal diameter of the maxillary sinus in the vertical plane) was defined as the longest distance from the lowest point of the inferior wall to the highest point of the superior wall on the sagittal image. The width of the maxillary sinus (maximal transverse diameter of the maxillary sinus in the horizontal plane) was defined as the longest perpendicular distance from the most prominent point of the medial wall to the most prominent point of the lateral wall on the axial image. The length of the maxillary sinus (maximal anteroposterior distance of the maxillary sinus in the sagittal plane) was defined as the longest distance from the most anterior point of the anterior wall to the most posterior point of the posterior wall on the axial image.

**Figure 1:**
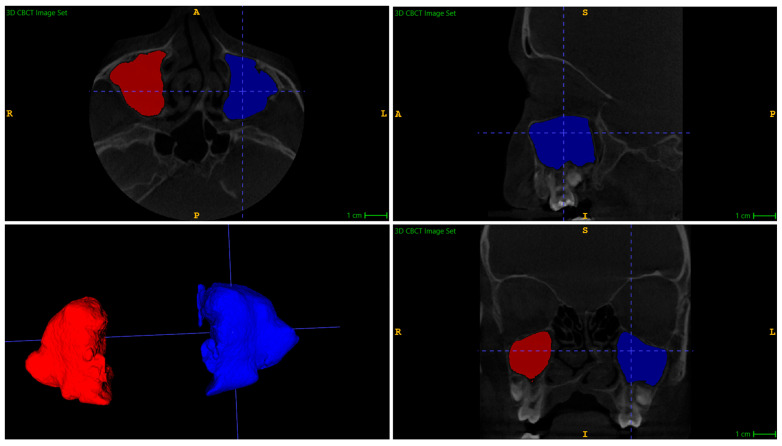
Maxillary sinus volume reconstruction in ITK-SNAP.

The volumes of the maxillary sinuses were measured before and after distraction using the ITK-SNAP version 4.2.2 (Philadelphia, PA, USA) 3D imaging software package. This software is a free, open-source segmentation tool used in neuroimaging and biomedical imaging research. The semi-automatic segmentation method was utilised to measure the maxillary sinus volumes, and the example of segmentation is shown in Figure 1. The surrounding tissues of all paranasal sinuses were removed using the Active Contour (Snake) Segmentation Mode, and pre-segmentation was performed using the Thresholding tool. Segmentation and measurement of the maxillary sinus were performed based on the bony contours of the sinuses. In cases with radiological features of chronic sinusitis, segmentation and measurement included the air-filled part of the sinus cavity, as well as any soft tissues within the skeletal boundaries of each sinus. The presence of soft tissues inside the cavity did not influence the anatomical volume or the reported measurements. Automatic software measurements of the sinus volumes (in cubic millimetres) were exported to a data table.

The volumetric parameters analysed were single sinus volume and total sinus volume. The single maxillary sinus volume was obtained as the volumetric measurement for an individual sinus, while the total sinus volume was defined as the sum of the volumetric measurements of the right and left maxillary sinuses of each patient.

The means, medians and standard deviations for all assessed parameters before and after distraction were calculated. The normality of the distribution of continuous variables was assessed using the Shapiro–Wilk test. The analysed data met the criteria to be considered as normally distributed. The reliability of the measurements was assessed by calculating intraclass correlation coefficients (ICC), a paired *t*-test was performed to evaluate changes in the parameters, and Pearson’s correlation coefficient was calculated to estimate the relationships between the distraction distance and volumetric changes in the sinus in the pre- and postoperative measurements.

The statistical analysis was performed with the Statistical Package for the Social Sciences, version 30.0.0 (SPSS Inc., Chicago, IL, USA). The level of significance was set at *p* < 0.05.

## 3. Results

Ten patients aged 13–20 years who met all the inclusion criteria were included in the study (four females and six males). Five patients presented bilateral cleft lip and palate (BCLP), and five presented unilateral cleft lip and palate (UCLP). Of the UCLP group, four had right-side clefts and one had a left-side cleft. All patients presented class III malocclusion with GOSLON Yardstick grade 4 or 5. The included patients had permanent dentition, indicating that the sinus volume was no longer increasing due to tooth eruption.

The results of maxillary distraction osteogenesis are presented for the operated group of patients with regard to cephalometric skeletal measurements, 3D CT sinus volume changes and linear measurements. The average time of the follow-up CT scan was 12.3 ± 6.98 months.

### 3.1. Skeletal Cephalometric Analysis

All patients presented class III malocclusion with GOSLON Yardstick grade 4 or 5 before the operation. The Wits appraisal (distance between perpendicular lines drawn from point A (on the maxilla) and point B (on the mandible) to the occlusal plane) ranged from −4.7 mm to −29.1 mm, and the ANB angle from −2.7° to −15.8°. The maxilla position was assessed via the SNA angle, and varied from 68.8° to 83.3°.

The SNA value increased by 9.27° ± 6.78° on average. The mean difference in the ANB angle of 9.88° ± 5.35° demonstrated forward movement of the maxilla during the distraction process. The mean ANB before the distraction was −8.04°, while that after surgery was 1.84°. The increase in Wits appraisal to 9.33 mm ± 7.93 mm corresponded with the ANB angle changes, with the Wits appraisal changing from −12.1 mm to −2.77 mm on average. There was a reduction in the SNB angle in 7 out of 10 patients, which corresponded with posterior rotation of the mandible. In the patients where the SNB angle increased, the mandible showed anterior rotation during the distraction process. Both of these changes were not statistically significant. The mean values of SN/NL, SN/ML and NL/ML showed increases of 1.68° ± 5.41°, 1.29° ± 4.49° and 2.57° ± 7.02°, respectively. This indicated posterior rotation of the maxillofacial complex, which corresponds with the distraction vector. The skeletal cephalometric analysis changes are presented in Table 1.

### 3.2. Maxillary Sinus Linear Measurements

Furthermore, linear measurements of the 20 sinuses were analysed. Regarding these measurements, although there were no statistically significant changes in sinus width or depth, a statistically significant increase in height was observed (*p* < 0.001), with a mean value of 4.46 ± 2.94 mm. Table 2 presents the linear changes in the maxillary sinuses.

### 3.3. Maxillary Sinus Volume

The sinuses were evaluated according to cleft type and laterality. The statistical analysis revealed that there was no statistically significant difference (*p* > 0.05) between the right and left sinuses, and the cleft side or type did not influence maxillary sinus volume. This allowed for the creation of a combined group of 20 sinuses, which strengthened the reliability of further statistical tests.

A total of 20 single sinus volumes were evaluated. The mean volume increase in a single maxillary sinus was 1983 ± 2889 mm^3^, which was a statistically significant change (n = 20, *p* = 0.003).

There was a statistically significant increase in total maxillary sinus volume (n = 10, *p* = 0.047), by 3965 mm^3^ ± 5456 mm^3^. The increase was greater in the BCLP group (n = 5), with a mean value of 3580 ± 4483 mm^3^, compared to 1577 ± 6846 mm^3^ in the UCLP group (n = 5); however, these differences were not statistically significant between the groups.

The data also revealed a moderate positive correlation between the distraction distance and single sinus volume change (r = 0.645, *p* = 0.001).

Table 3 shows the volumetric changes in the maxillary sinuses.

## 4. Discussion

This study evaluated changes in maxillary sinus volume following Le Fort I maxillary distraction with an extraoral appliance in a group of cleft patients in whom maxillary sinus growth was complete, all of which presented full permanent dentition [2]. Statistically significant increases in single sinus volume (n = 20) and the summarised volume of both sinuses for each patient (n = 10) were observed in the analysed group. The analysis revealed a positive correlation between the distraction distance and the change in volume of a single maxillary sinus: the greater the maxillary advancement, the more substantial the volume change observed.

The cephalometric measurements obtained in this study are consistent with the findings of other authors. Predictable anteroposterior correction of maxillary retrusion was achieved following the Le Fort I distraction osteogenesis procedure [26,27].

**Figure 2:**
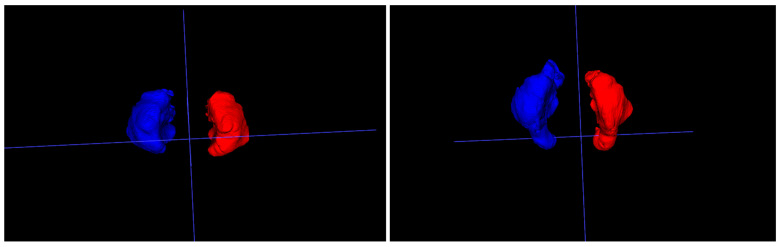
Maxillary sinus volume change before (**left**) and after (**right**) distraction.

The width and length measurements after the distraction osteogenesis did not change significantly. Height was the only measurement that increased in every patient following the procedure. Three-dimensional reconstruction of the maxillary sinuses after distraction osteogenesis revealed the formation of a new alveolar recess of the maxillary sinus, as illustrated in Figure 2. This newly formed area is assumed to be responsible for the overall increase in maxillary sinus volume and sinus height. A possible explanation is that the distraction vector was directed forward and downward, and the maxillary sinus height was measured along the maximal craniocaudal diameter in the vertical plane.

Although the authors did not find similar studies investigating sinus volume changes following distraction procedures, reliable studies on such changes after orthognathic procedures in both cleft and non-cleft patients are available. These studies suggest a decrease in maxillary sinus volume following maxillary advancement via traditional Le Fort I osteotomy, which is contrary to the findings of our study [28]. The osteotomy geometry and simultaneous maxillary impaction in advancement procedures may explain these differences [29,30]. Another variable that should be considered during bimaxillary surgery is the rotational component in the counter-clockwise direction, which may negatively influence the maxillary sinus volume after the surgery [31]. However, in the same study Koç et al. revealed that, in the group in which only advancement movement was performed, the maxillary sinus volume increased after the surgery [31]. During distraction osteogenesis, the distraction vector is usually directed forward and downward, which may suggest positive changes in the maxillary sinus volume after distraction.

In reference to the positive correlation between the distraction distance and the change in volume of a single maxillary sinus, the observed volume changes contrast with those reported in studies evaluating changes in the maxillary sinus volume after bimaxillary advancement surgeries [23,32]. However, the follow-up scan time differed in comparison to our study, and not only was the maxilla advanced but some impaction movement was also performed. Grab et al. reported 2 week and 6 month follow-up scans while, in our study, the average follow-up CT scan was performed at 12.3 ± 6.98 months [32]. The amount of movement performed on the maxilla during distraction is greater than that planned during bimaxillary surgeries. Moreover, some postoperative relapse in the consolidation phase after the distraction procedure is expected. Therefore, hypercorrection should be performed in the active phase, which may further influence the results. In contrast, when planning bimaxillary surgeries, the maxilla is positioned in the definite position and no hypercorrection is performed.

The authors observed that the semiautomatic method was less efficient during the segmentation process when radiological features of chronic sinusitis were presented before and after the distraction procedure, and it was challenging to properly adjust the contrast in the threshold mode in the maxillary sinuses during measurements where any inflammatory tissues were observed. The software used in this study allows for automatic and manual contrast adjustment [33]. The segmentation process took more time, compared to that for unaffected sinuses, but segmentation within bony boundaries of the sinus was still possible and the results obtained with the presented method were accurate. On the other hand, when the sinuses were fully air-filled, the software segmented them quickly and without errors. These findings did not affect the overall results of the study, as the maxillary sinus volume measurements were based on the bony contours, and the presence of soft tissue does not influence the total volume of the anatomical cavity. However, these observations may impact the choice of method used to most accurately measure the maxillary sinus volume in each patient. According to Gomes et al., the software used in this study remains accurate when evaluating irregularly shaped structures [10].

The authors point to a high risk of bias in the presented results as they were obtained from a small group of patients, which is mainly due to the rarity of the procedure and insufficient follow-up among some of the patients. Moreover, the studied group was not homogenous regarding the age of the patients, advancement amount and cleft type. Finally, a proper control group was not included.

## 5. Conclusions

Extraoral bone-anchored midface distraction osteogenesis was found to lead to statistically significant increases in the single sinus volume, total sinus volume and sinus height in a cleft cohort. These volume changes were not correlated with the patients’ sex or cleft type. Sinus volume changes occurred in addition to the correction of maxillary retrusion and improvement of the facial profile. In view of the small cohort analysed in this study and its non-uniform characteristics, further studies with larger, more homogenous cohorts are required for validation of the obtained results.

## Figures and Tables

**Table 1:** Comparison of reference points in Segner–Hasund cephalometric analysis.

		Before Distraction		After Distraction		Change	
	N	Mean	SD	Mean	SD	Mean	SD
SNA	10	76.35	4.89	85.62	9.16	9.27	6.78
SNB	10	84.43	6.24	83.78	7.24	−0.65	2.52
ANB	10	−8.04	4.32	1.84	4.06	9.88	5.35
WITS	10	−12.10	7.01	−2.77	4.05	9.33	7.93
SN/NL	10	5.19	2.93	6.87	5.93	1.68	5.41
SN/ML	10	33.14	5.79	34.43	7.20	1.29	4.49
NL/ML	10	28.67	7.80	31.24	8.93	2.57	7.02

SNA, sella–nasion–point A angle; SNB, sella–nasion–point B angle; ANB, point A–nasion–point B angle; WITS [Wits appraisal], distance between perpendicular lines drawn from point A (on the maxilla) and point B (on the mandible) to the occlusal plane; SN/NL, sella–nasion to nasal line (spina nasalis anterior–pterygomaxillare) angle; SN/ML, sella–nasion to mandibular line (gonion–gnathion) angle; NL/ML, nasal line (spina nasalis anterior–pterygomaxillare) to mandibular line (gonion–gnathion) angle.

**Table 2:** Linear changes in the maxillary sinuses.

Before Distraction				After Distraction				Change	Effect Size		
	N	Mean	SD	Mean	SD	Mean	SD	*p*	Cohen’s d	CI 95% (Effect Size)	
										Lower	Upper
MSW mm	20	27.7	5.42	27.5	8.08	−0.254	4.25	0.792	0.06	−0.380	0.498
MSD mm	20	39.2	5.96	40.0	7.36	0.8	3.17	0.273	0.253	−0.196	0.695
MSH mm	20	36.4	9.30	40.8	9.58	4.46	2.94	<0.001 *	1.52	0.860	2.16

MSW, maxillary sinus width; MSD, maxillary sinus depth; MSH, maxillary sinus height; * *p* < 0.05 in Paired Samples T-Test; CI 95%, 95% Confidence interval for an effect size.

**Table 3:** Volumetric changes in the maxillary sinuses.

Before Distraction After Distraction Change									Effect Size		
	N	Mean	SD	Mean	SD	Mean	SD	*p*	Cohen’s d	CI 95% (Effect Size)	
										Lower	Upper
RMSV mm^3^	10	17,182	6.793	18,750	7.550	1.568	1.990	0.034 *	0.788	0.056	1.49
LMSV mm^3^	10	17,220	6.823	19,618	8.772	2.398	3.644	0.067	0.658	0.0449	1.33
TMSV mm^3^	10	34,402	13.541	38,368	16.108	3.965	5.456	0.047 *	0.727	0.0089	1.41
SMSV mm^3^	20	17,201	6.627	19,184	7.978	1.983	2.889	0.003 *	0.767	0.258	1.26

RMSV, right maxillary sinus volume; LMSV, left maxillary sinus volume; TMSV, total maxillary sinus volume; SMSV, single maxillary sinus volume; * *p* < 0.05 in Paired Samples T-Test; CI 95%, 95% Confidence interval for an effect size.

## Data Availability

All data are provided within the manuscript.
